# Factors affecting the use of clinical practice guidelines by hospital physicians: the interplay of IT infrastructure and physician attitudes

**DOI:** 10.1186/s13012-020-01056-1

**Published:** 2020-11-25

**Authors:** Noriko Sasaki, Naohito Yamaguchi, Akiko Okumura, Masahiro Yoshida, Hiroyuki Sugawara, Jung-ho Shin, Susumu Kunisawa, Yuichi Imanaka

**Affiliations:** 1 Department of Healthcare Economics and Quality Management, Kyoto University Graduate School of Medicine, Yoshida Konoe-cho, Sakyo-ku, Kyoto, 606-8501 Japan; 2 Japan Council for Quality Health Care, 1-4-17, Toyo Bldg., Kandamisaki-cho, Chiyoda-ku, Tokyo, 101-0061 Japan; 3 Saiseikai Research Institute of Health Care and Welfare, 1-4-28 Mita International Bldg 21st Floor, Mita, Minato-ku, Tokyo, 101-0061 Japan

**Keywords:** Clinical practice guidelines, Physician education, Digital preference, Hospital IT infrastructure, Habitual behaviours, Implementation

## Abstract

**Background:**

Compliance with clinical practice guidelines (CPGs) remains insufficient around the world, despite frequent updates and continuing efforts to disseminate and implement these guidelines through a variety of strategies. We describe the current status of young resident physician practices towards CPGs and investigate the multiple factors associated with the active use of CPGs, including the physician’s knowledge, attitudes, behaviours, CPG-related education received, and the hospital’s IT infrastructures. The aim is to identify a more effective point for intervention to promote CPG implementation.

**Methods:**

We conducted a questionnaire survey among resident physicians working at 111 hospitals across Japan in 2015 and used results with hospital IT score data collected from a prior survey. Multivariable logistic regression analysis was performed to examine the determinants of frequent use of CPGs (defined at least once per week). The independent variables were selected based on physician demographics, clinical speciality and careers, daily knowledge and behaviour items, CPG-related education received, digital preference, and hospital IT score (high/medium/low), with and without interaction terms.

**Results:**

Responses from 535 resident physicians, at 61 hospitals, were analysed. The median hospital IT score was 6 out of a possible 10 points. Physicians who had learned about CPGs tended to work at hospitals with medium to high IT scores, had easier access to paywalled medical databases, and had better knowledge of the guideline network ‘Minds’. In addition, these physicians tended to use CPGs electronically. A physician’s behaviour towards using CPGs for therapeutic decision-making was strongly associated with frequent use of CPGs (odds ratio [95% CI] 6.1 [3.6–10.4]), which indicated that a physician’s habit strongly promotes CPG use. Moreover, CPG-related education was associated with active use of CPGs (OR1.7 [1.1–2.5]). The interaction effects between individual digital preferences and higher hospital IT score were also observed for frequent CPG use (OR2.9 [0.9–8.8]).

**Conclusions:**

A physician’s habitual behaviours, CPG-related education, and a combination of individual digital preference and superior hospital IT infrastructure are key to bridging the gap between the use and implementation of CPGs.

**Supplementary information:**

**Supplementary information** accompanies this paper at 10.1186/s13012-020-01056-1.

Contributions to the literature

- Despite frequent updates and continuing efforts to implement clinical practice guidelines (CPGs) through a variety of strategies, compliance with CPGs remains insufficient.
- A physician’s habitual behaviours, CPG-related education, and a combination of individual digital preference and superior hospital IT infrastructures, were identified as factors affecting the physician’s use of CPGs.
- This study clarifies the interacting mechanisms between a physician’s digital preferences and usability; such clarification should contribute to the next generation of conceptual frameworks for the effective implementation of CPGs, as individual physicians are the direct users of CPGs and will be the link between IT infrastructure and quality of care.

## Background

Compliance with clinical practice guidelines (CPGs) is still reported to be insufficient around the world, despite the frequent updating of CPGs and continuing efforts to disseminate and implement them through a variety of strategies [1–5]. Systematic strategies to implement CPGs into daily medical practice have been developed over several decades. For example, national systems of CPGs and standards have been established for those developing guidelines and for professional users, as well as the general public, and both the effective use of clinical information systems and continuing professional education for health professionals have been promoted in many countries [1–6].

In the field of implementation science, strategies required for implementation should take into account not a single but multifaceted approach, such as at the individual, organizational, system, and policy levels, and it is recommended that relevant conceptual frameworks should be applied for effective CPG implementation [1, 7–9]. However, multiple barriers to implementation have been identified, including uncertainty, professional norms, and contextual factors such as whether the setting involves chronic or acute diseases. Furthermore, in the context of adapting appropriate models for the development of successful strategies in such a complex environment, the requirement of an inter-relational process between stages and the many different levels of actors and organizations at multiple intervention points have often resulted in a failure to implement guidelines in general practice [1, 6, 10–12].

With regard to *physician-level* factors, a number of early studies showed that while the effectiveness of passive methods (e.g. didactic lectures, publication of consensus statements, mass mailings) was low, active methods, including continuing medical education and interactive education with professionals, tended to be useful in changing physician behaviour [1, 6, 13, 14]. Subsequent research in healthcare quality and evidence-based medicine, however, placed the focus on the role of organizational structures and policies from a *system-level* ‘total quality management’ perspective [1].

Moreover, the rapid evolution of information technology (IT) in recent decades has led to an increasingly high quality (i.e. availability and usability) of hospital IT infrastructure, such as wireless local area networks (LAN) and extensive medical evidence databases. This can play a crucial role in CPG use at the physician level, as easy and timely accessibility to the latest CPGs will encourage their routine use. In fact, previous studies have reported the importance of institutional equipment, technological capital, and accessibility to guideline-related resources, not to mention individual awareness, usability, and acceptance of the contents [13–15].

We recently reported that hospitals with superior IT infrastructure tended to have higher adherence to CPGs for perioperative antibiotic prophylaxis and that a high level of access to commercial medical evidence databases and accessibility to the Internet were key factors for the quality of care in acute care hospitals [16]. However, it remains unclear how physician-level knowledge, behaviours, and digital preferences, as well as system-level factors such as hospital IT infrastructure, relate to active use of CPGs in general.

In Japan, more than 270 updated CPGs have been assessed and disseminated by the government-funded Medical Information Network Distribution Service (*Minds*) Guideline Centre [17] since 2003, but the actual use of these CPGs by physicians in their daily practice remains unknown.

In this study, we sought to clarify the nature and extent of current CPG use by young physicians in the ‘Digital Era’ and to investigate the factors associated with the active use of CPGs, taking into account the individual’s knowledge, attitudes, behaviours (including habits), CPG-related education received, and hospital IT infrastructure—all in an effort to identify a more effective point for intervention to promote the implementation of CPGs.

## Methods

### Data sources

As part of the *Minds* national activities, the Minds-QIP collaborative project was carried out between 2014 and 2018, with the objective of effectively disseminating and implementing CPGs throughout Japan. This survey study is embedded within this wider Minds-QIP research. Regarding the Quality Indicator/Improvement Project (QIP), it is a project which intends to improve clinical performance in acute care hospitals across Japan and generates clinical and economic performance indicators since 1995. More than 500 QIP participating hospitals voluntarily submit administrative data, and the project communicates with hospitals through periodic reporting activities and occasional surveys [18, 19].

The survey described here was conducted between February and May 2015. Questionnaires were mailed to the resident physicians at QIP member hospitals. Survey results, together with infrastructure ratings data generated from a previous hospital-level survey conducted in 2015 [16], formed the basis of our analysis.

The questionnaire included items on the knowledge, attitudes, and behaviours of resident physicians regarding evidence-based practices and hospital IT infrastructure, as well as the actual provision of IT infrastructure by the hospitals in which the residents practised (including LAN deployment and usability of medical evidence databases; the details are to be mentioned later).

### Physicians’ survey

The survey questionnaire was developed based on a literature review and semi-structured, face-to-face interviews with hospital administrators, IT directors and/or education directors (*n* = 15), and focus group interviews and pre-testing with junior and senior resident physicians (*n* = 52) from five major teaching hospitals. The initial two-page survey was reviewed and refined for content validity by the Minds-QIP working group, which included six senior researchers, three of whom were practising physicians. The questionnaire was finalized and then validated by experts on the committee for practice guideline implementation.

We used purposive sampling to collect our data. Junior and senior resident physicians working in acute care hospitals were targeted regardless of their specialties. Since we could not directly access each physician, we asked hospital administrators to encourage resident physicians to participate using a pre-notification letter to promote the survey. However, physician participation was wholly voluntary. In order to optimize the response rate, we used proven methods such as including a stamped return envelope and sending follow-up mail with the questionnaire. We made every effort to keep the questionnaire short and to make it interesting and user friendly.

Physician knowledge, attitudes and behaviours were measured through the items shown in the table in Additional file 1. With respect to physician attitudes, we considered the preferred mode of CPG delivery to be not merely a preference but a key attitude for the requirement of high-quality IT infrastructure. Physician behaviours regarding their active use of the guidelines were measured to explore the multifaceted aspects of daily practice settings, including the physician’s habits of guideline use.

### Hospital IT scores to identify hospital IT infrastructure promoting CPG use

In order to assess hospital IT infrastructure, an IT score for each hospital was calculated using the results from the prior hospital-level survey noted above [16].

A hospital IT score (out of a possible 10 points) was calculated for each hospital, based on the checklist shown in Table 1. The checklist was designed to focus on the following three elements of hospital IT infrastructure: (1) accessibility to the Internet and other information sources, including wired/wireless LAN availability; (2) access to paywalled medical evidence databases in English and Japanese; and (3) medical library and intranet usability, such as the availability of a well-organized intranet interface and activities for improving the medical library [16].

**Table 1:** Checklist (audit tool) for assessing hospital IT infrastructure

1. What information sources are available in your hospital? (Multiple answers allowed)	
□ Igaku Chuo Zasshi (ICHUSHI) Medical Literature Database [Hospital Subscription] [1]^a^	
□ Charged database in English including UpToDate®, Clinical Key®, Ovid®, DynaMed® [Hospital Subscription] [1]	
□ No charged database is provided in the hospital IT system [0].	
2. Where are the major locations in your hospital for Internet use with wired LAN access? (Multiple answers allowed)	
□ Outpatient clinics [1]	
□ Wards [1]	
□ Libraries [0]	
□ Medical offices [0]	
3. Is wireless LAN available in your hospital?	
□ Yes, available with no limitations [2].	
□ Yes, with limited access points [1].	
□ No, not available. (Only available by individual or medical office subscription) [0].	
4. Does your hospital provide an intranet homepage with user-friendly interface in order to easily access digital libraries including various medical journals?	
□ Yes [1]	
□ No [0]	
5. Which applies to your hospital regarding medical library activities? (Multiple answers allowed)	
□ Periodic meetings held to improve the information retrieval environment [1].	
□ Continuously working to improve library services and usability (e.g., promoting paperless movements) [1]	
□ Participation in hospital librarian associations and communication with other hospital librarians [1]	
□ Nothing in particular [0].	
**Total score /10**	

^a^[ ] Scores for ‘Yes’

### Statistical analysis

We examined the behaviour of resident physicians in relation to their active use of guidelines and investigated such potentially influencing factors as the resident’s knowledge, attitudes, and CPG- related education received, as well as the available IT infrastructures in the workplace.

Descriptive statistics were presented to show hospital IT score categories (low, 0 to 3 points; medium, 4 to 6 points; high, 7 to 10 points). Multivariable logistic regression analysis was then performed to identify the determinants of frequent use of CPGs (defined at least once per week). The independent variables for the two models that were considered were selected based on physician demographics, clinical speciality and careers, daily knowledge and attitude items, CPG-related education received, digital preferences, and hospital IT score (medium to high, or low). The forced entry method was used to incorporate the aforementioned independent variables into model A. A similar approach was used for model B, which included not only the aforementioned variables, but also an interaction term for ‘preference to use digital guidelines’ and ‘medium-to-high hospital IT score’ (Table 4).

Multivariable logistic regression was performed using the PROC LOGISTICS procedure in SAS software version 9.4 for Windows (SAS Institute Inc, Cary, NC); other statistical calculations were done with IBM SPSS Statistics for Windows, Version 23.0 (IBM Corp., Armonk, NY). All tests were two-tailed, with a significance level of 0.05.

## Results

### Baseline characteristics by hospital IT infrastructures

**Fig. 1:**
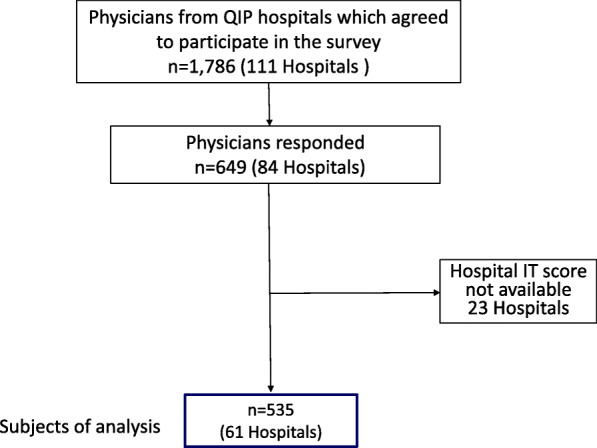
Flow diagram of the respondents’ selection process

A total of 649 residents from 84 hospitals responded to the questionnaire (response rate: 36.3%); 535 residentsfrom 61 hospitals were included in the final analysis (Fig. 1). (Twenty-three hospitals did not respond to the hospital-level survey, which made it impossible to calculate their IT scores. Consequently, these hospitals were excluded from our analysis.) Descriptive statistics for the hospitals and respondents by hospital IT score categories are shown in Table 2. The median hospital IT score (interquartile range, IQR) was 6 (4–8) points out of apossible 10 points. More than 85% of the hospitals were teaching hospitals and more than 85% of the respondents were 20 to 39 years of age in hospitals with a medium to high IT score. Twenty-seven percent of all respondents majored in internal medicine.

**Table 2:** Baseline characteristics of hospitals and physicians

	Hospital IT score			Overall
	Low (0-3)	Medium (4-6)	High (7-10)	
**Hospitals**	*N* = 16	*N* = 28	*N* = 17	*N* = 61
Beds, median (IQR)	336 (211-492)	396 (255-513)	535 (412-648)	425 (296-529)
Teaching hospitals, *N* (%)	11 (68.8)	24 (85.7)	15 (88.2)	50 (82.0)
Hospital IT score, median (IQR)	3.0 (2.0-3.0)	5.0 (4.0-6.0)	8.0 (7.0-8.0)	6.0 (4.0-8.0)
**Physicians** (*n*, %)	*n* = 84	*n* = 247	*n* = 204	*n* = 535
Sex				
Male	61 (72.6)	190 (76.9)	158 (77.5)	409 (76.4)
Female	23 (27.4)	57 (23.1)	46 (22.5)	126 (23.6)
Age				
20~29 years	56 (66.7)	173 (70.0)	151 (74.0)	380 (71.0)
30~39 years	13 (15.5)	42 (17.0)	26 (12.7)	81 (15.1)
40~49 years	1 (1.2)	4 (1.6)	3 (1.5)	8 (1.5)
50~59 years	0	0	1 (0.5)	1 (0.2)
N/A	14 (16.7)	28 (11.3)	23 (11.3)	65 (12.1)
Years of residency				
Junior (1 to 2 years)	48 (57.1)	157 (63.6)	124 (60.8)	329 (61.5)
Senior (3 to 5 years)	19 (22.6)	57 (23.1)	51 (25.0)	127 (23.7)
Specialty (include rotations in the residency programmes)				
Internal medicine	21 (25.0)	74 (30.0)	48 (23.5)	143 (26.7)
Surgery	17 (20.2)	55 (22.3)	32 (15.7)	104 (19.4)
Emergency	4 (4.8)	8 (3.2)	10 (4.9)	22 (4.1)
Paediatrics	2 (2.4)	8 (3.2)	13 (6.4)	23 (4.3)
N/A	40 (47.6)	102 (41.3)	101 (49.5)	243 (45.4)

*IQR* interquartile range, *N/A* not available

Table 3 shows the differences in the respondents’ practices and perceptions, including their knowledge and attitudes, categorized by hospital IT infrastructures. Resident physicians who worked at hospitals with a medium to high IT score tended to be more familiar with the national guideline network ‘Minds’ than were those who worked at hospitals with a low IT score. Similarly, more resident physicians who had learned about CPGs tended to work at hospitals with a medium to high IT score and had easier access to paywalled medical databases, especially those in English. In addition, they tended to use practice guidelines electronically. The level of satisfaction with hospital IT infrastructure was also higher among resident physicians in hospitals with a medium to high IT score.

**Table 3:** Differences in respondents’ practices and perceptions by hospital IT score categories (*n* = 535)

*n* (%)	Hospital IT score			*p*^†^
	Low (0-3) *n* = 84	Medium (4-6) *n* = 247	High (7-10) *n* = 204	
*Available resources*				
Use of private electronic devices such as desktop PC, notebook, tablet, or smartphone for daily practice				
Yes	67 (17.1)	183 (46.7)	142 (36.2)	0.193
Subscription to paywalled medical databases				
English	38 (12.8)	126 (42.4)	133 (44.8)	0.001**
Japanese	36 (13.4)	118 (44.0)	114 (42.5)	0.081
Obstacles to use practice guidelines				
Unable to access to information	36 (15.4)	113 (48.3)	85 (36.3)	0.674
Unable to retrieve necessary information	26 (15.1)	79 (45.9)	67 (39.0)	0.950
*Education received*				
Experience of education related to practice guidelines				
At workplace or at medical school	48 (14.2)	148 (43.8)	142 (42.0)	0.048*
*Knowledge*				
Knowledge of the national guideline network, ‘Minds’				
Yes	20 (13.6)	82 (55.8)	45 (30.6)	0.022*
*Attitude*				
Major way to use practice guidelines				
Book	46 (19.1)	140 (44.4)	116 (36.5)	0.150
Electronic	55 (14.0)	192 (48.7)	147 (37.3)	0.071
*Behaviour*				
Frequency of using guidelines in daily practice				
At least once a week	49 (14.8)	158 (47.7)	124 (37.5)	0.604
When to use CPGs				
For therapeutic decision making (Yes)	74 (17.2)	200 (46.4)	157 (36.4)	0.093
To gain related knowledge (Yes)	64 (17.1)	177 (47.3)	133 (35.6)	0.129
For shared decision-making with patients (Yes)	18 (21.2)	42 (49.4)	25 (29.4)	0.124
*Satisfaction*				
Satisfied with their own hospital IT circumstances				
Yes	38 (13.4)	121 (42.8)	124 (43.8)	0.014*

*P < 0.05

**P < 0.01

^†^Three groups were compared using chi-squared test

### Factors associated with active use of clinical practice guidelines

Table 4 shows the results of the regression analysis. Male, CPG-related education, preference to use digital guidelines, and a behaviour to use CPGs for therapeutic decision-making were independently associated with active use of CPGs (model A). With respect to factors related to physician behaviours, using CPGs for therapeutic decision-making appeared to have the greatest influence (OR [95% CI] 6.2 [3.6–10.4]), followed by individual digital preference for using CPGs (OR 2.3 [1.5–3.7]). A higher hospital IT score alone showed no significant relationship with active use of CPGs. However, when considering the interaction effects between individual digital preference and higher hospital IT score, we found a higher tendency to actively use CPGs (OR 2.9 [0.9–8.8]: model B). No other factor showed interaction effects with higher hospital IT score. In addition, experience of CPG-related education was associated with greater active use of CPGs (OR 1.7 [1.0–2.5]), regardless of the model conditions.

**Table 4:** Determinants of active use of guidelines in physicians’daily practice

Variables	Adjusted odds ratio [95% CI]	
	Model A	Model B
Male (*ref* Female)	1.58 [1.04–2.40]*	1.59 [1.04–2.43]*
Senior residency (*ref* Junior residency)	1.47 [0.89–2.42]	1.47 [0.89–2.43]
Internal medicine specialty (*ref* Others)	1.40 [0.88–2.23]	1.41 [0.88–2.25]
Education for CPG use (*ref* no education)	1.72 [1.04–2.50]**	1.68 [1.12–2.53]*
Knowledge of guideline network 'Minds' (*ref* No knowledge)	1.00 [0.64–1.56]	0.97 [0.62–1.51]
Preference to use digital guidelines (*ref* book guidelines)	2.33 [1.45–3.74]***	1.01 [0.37–2.76]
When to use CPGs		
For therapeutic decision-making (Yes)	6.15 [3.62–10.43]***	6.09 [3.58–10.35]***
To gain related knowledge (Yes)	0.78 [0.49–1.24]	0.77 [0.48–1.22]
For shared decision-making with patients (Yes)	1.77 [0.97–3.22]	1.82 [0.10–3.31]
Hospital IT score_Medium to High (*ref* Low)	1.23 [0.72–2.12]	0.61 [0.24–1.53]
Preference to use digital guidelines x Hospital IT score_Medium to High	–	2.88 [0.94–8.83]
C-statistics	0.752	0.753

*CI* confidence interval, *CPG* clinical practice guidelines

Model A—without an interaction term, model B—with interaction terms

*P < 0.05

**P < 0.01

***P < 0.001

## Discussion

We found that resident physicians who actively use CPGs had prior knowledge of CPGs, preferred to use CPGs digitally at hospitals with well-equipped IT systems, or habitually used CPGs in therapeutic decision-making.

### Necessity for extensive education related to CPG use

According to our results, resident physicians who had received CPG-related education before or after becoming a physician used CPGs much more frequently (OR [95%CI] 1.7 [1.0–2.5]: Table 4) than those who had not received such education. To date, however, evidence-based practice and CPGs, from development to dissemination and implementation, have not been adopted as part of the basic curriculum for medical students in Japan. Rather, they are sporadically delivered only to those interested in the topic, through continuing medical education or activities undertaken by medical societies. For example, this may be done through courses on the history of evidence-based medicine, where one might learn how CPGs have evolved and become standardized, or how to develop clinical questions, or how to use internationally adopted methods including tools for guideline development and evaluation of guidelines such as Appraisal of Guidelines for Research & Evaluation II (AGREE-II) [20, 21] and the Grading of Recommendations, Assessment, Development and Evaluations (GRADE) [22]. Methods for using how to use evidence and CPGs effectively in daily practice should be taught as a specific part of the medical curriculum or as a systematic programme with a sufficient amount of time allotted to it.

Furthermore, there is need for digital skills’ training with an emphasis on digital literacy in the context of CPG implementation, which would include knowledge of the CPG clearing houses in various countries (e.g. the Scottish Intercollegiate Guidelines Network (SIGN), the ECRI Guideline Trust ^TM^, the Guidelines International Network’s International Guidelines Database, and the *Minds*). Knowledge of how to retrieve the latest digital content, including ways to use multiple search engines and paywalled medical datasets, and knowledge of health information systems, including various applications and technologies, are essential [23]. In today’s information-overloaded world, clinicians are expected to possess integrated skills across a number of fields rather than solely in health care, in order to keep up with the latest information [24, 25]

To note, physicians who used CPGs when they make therapeutic decision tended to have a much stronger association with the frequent use of CPGs (OR [95%CI] 6.2 [3.6–10.4]). On the other hand, physicians who used CPGs when they wanted to gain knowledge or when they made decisions with their patients had no significant association with the active use of CPGs. These results suggest that physicians who use CPGs when making therapeutic decisions use them as a matter of habit. Our results are in line with a previous systematic review indicating that habit plays a significant role in professional healthcare behaviour [26], lending support to our suggestion that habitual behaviour would be an important point of intervention in the context of CPG implementation. Since ‘there is no reliable way to choose strategies that are appropriate for implementing guidelines’ [24] thus far, identifying effective points or populations for intervention to promote CPG use would seem to be important when planning or creating a framework for implementation strategies [1, 7–9]. One approach would be to encourage physician to develop the habit of using CPGs in their routine decision making by providing educational programmes that promote such use during an earlier phase of their careers. In other words, physicians who would not habitually use CPGs in their therapeutic decision making would be a promising future target population for intervention.

In sum, in order to promote evidence-based practice, it is crucial to educate physicians in CPG-related areas including the development of digital skills and general digital knowledge, and to foster the clinical habit of using CPGs in the daily decision-making process.

### Individual digital preferences and IT infrastructures at hospitals

We found that the physician’s digital preferences were independently correlated with active use of CPGs (OR 2.3 [1.5–3.7]; Table 4: model A). Moreover, the combination of individual digital preference and a higher hospital IT score was associated with higher active use of CPGs (OR 2.9 [0.9–8.8]), while a higher hospital IT score alone showed no significant relationship with frequent CPG use (Table 4: model B). These results indicate that the poor quality of a hospital’s IT infrastructure may hinder a physician’s daily use of CPGs despite the physician’s preference for IT and possession of sufficient IT skills.

In a previous study, we reported that the provision of a LAN and access to the Internet and electronic health records is still limited in many hospitals in Japan [16], where 80% of the more than 8000 hospitals are privately owned and relatively small [27], despite the fact that provision of access to paywalled medical evidence databases and accessibility to the Internet were shown to be strong indicators of quality of care in larger or teaching hospitals [16]. However, we did not determine how these circumstances affected the physician’s daily activities with regard to the active use of CPGs.

The uniqueness of the present study centres on our development of a pilot tool to evaluate hospital IT infrastructure based on our previous study, and our use of this tool to measure with a single score the accessibility and usability of CPGs afforded by various multifaceted hospital IT infrastructures (Table 1). The adoption of various types of IT infrastructure in hospitals has been reported to be related with desirable patient quality outcomes [28, 29]. Our results are in line with the conceptual framework known as Human, Organization and Technology (HOT-fit), which is used to evaluate the implementation of health information systems, with a focus on the relative disposition of technology (system quality/information quality/service quality), human (system use/ user satisfaction), and organization (structure/environment) [30, 31]. Although several recent studies using this framework have targeted the effective implementation of a guideline-based Clinical Decision Support System (CDSS) and electronic medical record systems [2, 31, 32], they provide no practical or quantitative information related to physician attitudes and behaviours, including their digital preferences and their interactions with hospital IT infrastructure. In addition, despite the fact that a number of conceptual frameworks intending to refine implementation strategies have been reported, few studies have highlighted the interactions between individual-level factors and system-level factors.

Behaviour change theory makes it clear that education or information aimed at increasing a physician’s knowledge is, in itself, insufficient to change behaviour. According to the ‘Theoretical Domain Framework’, which provides an integrative model to assess barriers to behavioural changes in achieving implementation, our setting fits perfectly with the ‘Person×environment action’ construct in the ‘Environmental context and resources’ domain, and with the ‘Routine/automatic/habit’ construct in the ‘Nature of the behaviours’ domain [10–12]. However, our results become more understandable in the larger context of ‘Systems thinking’ [33, 34], in that, no single model or combination of several models can determine the optimal intervention point for implementation due to the inter-relational process between stages. Clearly, many different levels of actors and organizations for multiple intervention points need to be taken into account, when formulating strategies to implement the guidelines.

Our new findings thus offer a valuable input for the next generation of conceptual frameworks, taking into account the interactions between physicians and hospital IT infrastructure. Our results also suggest that hospital administrators, clinicians and researchers should all be aware of both physician-level and system-level digital factors related to the effective implementation of CPGs.

### Implications for daily clinical practice

Clinicians often have limited time to search for and retrieve medical information and typically have only a limited IT infrastructure at their workplace. Our study revealed the interaction effects between a physician’s individual digital preferences and a well-equipped IT infrastructure are associated with the active use of CPGs. Ideally hospital administrators should invest in IT infrastructure that facilitates the accessibility and usability of CPGs in order to narrow the existing evidence–practice gaps. However, not all hospitals have the financial resources for such an IT infrastructure. Recently, there have been significant international movements to create seamless digital guideline platforms that connect actors across the field of evidence-based health care, including primary researchers (evidence producers), those who summarize research into systematic reviews (evidence synthesizers), those who create clinical practice guidelines and decision aids (evidence processors and disseminators) and those responsible for implementing and evaluating evidence to improve health care (evidence implementers)—the so-called evidence ecosystem [35]. As such, gathering evidence-based knowledge and the construction of information platforms beyond individual hospitals, regardless of financial constraints, may be part of the solution to effectively implement CPGs. In this context, our results suggest that CPG-related education, including digital skills education, may help promote CPG use. Considering the decades-long accumulation of implementation science and CPG-related knowledge and methods [1, 15, 20–22], it may be possible to create an information sharing system to expand worldwide knowledge and make these resources available to young physicians and medical students straight away in an ecological manner.

There are some potential limitations to this study. First, the respondents were young resident physicians; therefore, we need to be cautious when extrapolating the results to the wider medical profession, including senior and older physicians. Due to the ‘digital divide’, older people generally have lower digital search skills [36], so older physicians may prefer to use printed CPGs, and this thus limits the generalizability of our results. In fact, in an earlier survey, we found a higher proportion of printed CPG adoption among hospital administrators [16]. Second, the low response rate in our survey could increase the likelihood of response bias in favour of more highly motivated physicians who use the guidelines in relatively large hospitals. However, the response rate in physician survey has historically been rather low, the response rate in our study seems comparable to the rates in previous studies [37, 38]. Third, because of the self-reporting nature of the questionnaire, behaviours should be interpreted with caution since a socially desirable responding, induced by such factors as the tendency to want to present a favourable image of oneself on a questionnaire, could be present. However, our questionnaire items did not include particularly sensitive questions, especially when compared to previous research in such areas as dietary intake, domestic violence, and sexual practices. Thus, any such bias is unlikely to have materially affected our results. Fourth, we could not conduct a multilevel analysis because of a convergence failure. The number of samples per cluster might be one of the reasons for this. Fifth, hospital IT scores mainly focused on the adequacy of IT infrastructure for medical information retrieval in relation to accessibility to the latest practice guidelines. This may reflect only a part of the IT infrastructure rather than the IT system as a whole, which would include electronic health record adoption, the use of an electronic prescription system, and automated reminders. Lastly, we could not identify the organizational climate or culture, such as readiness for change, or the affordability of IT infrastructure at each hospital. Additional study would be needed to examine the effect of such factors [1]. Despite these limitations, our study recognizes that individual physicians are the direct users of CPGs and are likely to be the link between IT infrastructure and quality of care. Accordingly, we provide a detailed clarification of the interacting mechanisms between physician attitudes and digital usability.

In terms of implementation strategies, physicians who do not habitually use CPGs in therapeutic decision-making should be a future target population for intervention. Moreover, improving CPG-related education and hospital IT infrastructure that take into account the digital preference of physicians may enhance actual CPG use.

## Conclusions

The habitual behaviours of physicians, CPG-related education, and a combination of individual digital preference and superior hospital IT infrastructure are key to bridging the gap between the use and implementation of CPGs.

## Supplementary information

**Additional file 1.** Table. Original core questions

**Additional file 2.** STROBE checklist

## Data Availability

Please contact author for data requests.

## Abbreviations

CPG: Clinical practice guideline
IT: Information technology
LAN: Local area networks
Minds: Medical Information Network Distribution Service
OR: Odds ratio
QIP: Quality Indicator/Improvement Project
