# Supplementary material for: Factors affecting the use of clinical practice guidelines by hospital physicians: the interplay of IT infrastructure and physician attitudes
Source: Implement Sci. 2020 Nov 25;15:101. doi: 10.1186/s13012-020-01056-1 (PMC7687727; doi:10.1186/s13012-020-01056-1)
Supplement: Supplementary file 1 — Additional file 1. Table. Original core questions [file 13012_2020_1056_MOESM1_ESM.docx]

Additional file 1_Table. Original core questions

Q1. What information sources are available in your hospital? (Please choose all options that would apply)

□ 1. PubMed □2. Google/Yahoo □3. Pharmaceutical Package Inserts □ 4. Pharmaceutical Interview Forms

□ 5. Scientific Society Websites □ 6. MINDS

□ 7. *Igaku Chuo Zasshi* (ICHUSHI) Medical Literature Database [**Hospital** Subscription]

□ 8. *Igaku Chuo Zasshi* (ICHUSHI) Medical Literature Database [**Medical Office** Subscription]

□ 9. UpToDate [**Hospital** Subscription] □ 10. UpToDate [**Medical Office** Subscription]

□ 11. Cochrane Review 　□ 12. ClinicalKey 　□13. Ovid 　　□ 14. DynaMed

□ 15. *Today’s Diagnosis* [**Electronic Edition**] □ 16. *Today’s Therapy* [**Electronic Edition**]

□ 17. Various Clinical Practice Guidelines [**Electronic Versions**]

□ 18. *Today’s Diagnosis* [**Print Edition**]　　 □ 19. *Today’s Therapy* [**Print Edition**]

□ 20. Various Clinical Practice Guidelines **[Print Versions**] 　　□21. Other

Q2-1. Do you use private electronic devices such as desktop PC, notebook, tablet, or smartphone for daily practice?

□Yes □No

Q2-2. Is *wireless* LAN available in your hospital?

□Yes, available with no limitations. □Yes, with limited access points. □ No, not available.

Q3. Are you satisfied with the IT infrastructure in your hospital?

□ Yes □ No □ Other

Q4. How frequent do you use clinical practice guidelines in your daily clinical settings? (If you choose answer 1 to 4 here, please answer Q5-1 to Q5-4.)

□1. Almost every day □2. More than once a week □3. More than once a month □4.More than once a year

□5. Rarely □6. Never

Q5-1. What specialty area of clinical practice guidelines do you usually use in daily practice? (Please choose all options that apply)

□ 1. Medical oncology □2. Neurology □3. Orthopedics □ 4.Cardiology □ 5. Respiratory medicine

□ 6. Gastroenterology □7. Dentistry □ 8.Nephrology □ 9.Endocrinology/Metabolism

□10. Allergy/ Autoimmune disease □11. Dermatology/Ophthalmology/Otolaryngology □12.Obstetrics/ Gynecology □13.Pediatrics □14.Mental health □15. Infectious diseases □16. Health checkup/Preventive health care

□17. Emergency medicine □18. Other

Q5-2. Which applies to your daily practice in using clinical practice guidelines?

□ I mainly use practice guidelines of the specialty area which is currently my major area.

□ I mainly use practice guidelines of the specialty area which is not currently my major area.

Q5-3. In which of the following settings do you often use the guidelines? *(Multiple answers allowed.)*

□ When determining treatment strategies

□ To acquire knowledge

□ For shared decision-making with patients

Q5-4. Please state your main browsing methods for clinical practice guidelines *(Multiple answers allowed.)*

□ Printed paper

□ Online

□ Downloaded digital files

Q6. Are you aware of the website of the national guideline network ‘Minds’?

□ Yes □ I’ve seen it, but I’m not clear about the content. □ No

Q7-1. Have you ever received education related to the clinical practice guidelines during residency in your hospital?

□ Yes □ No □ Don’t know

Q7-2. Did you receive education related to clinical practice guidelines when you were a medical student?

□ Yes □ No □Don’t know

Q7-3. What do you think are the reasons that give rise to difficulties in using the clinical practice guidelines? *(Multiple answers allowed.)*

□ Uncertainty regarding how to use the guidelines

□ Inability to access information

□ Ability to access information in general, but inability to acquire necessary information

□ Dissatisfaction with the information on clinical practice guidelines

□ Other
